# Stable isotope evidence for the Bottom Convective Layer homogeneity in the Black Sea

**DOI:** 10.1186/1467-4866-15-3

**Published:** 2014-04-17

**Authors:** Alexander V Dubinin, Elena O Dubinina, Tatyana P Demidova, Nataliya M Kokryatskaya, Maria N Rimskaya-Korsakova, Sofia A Kosova, Evgeniy V Yakushev

**Affiliations:** 1 PP Shirshov Institute of Oceanology, Russian Academy of Sciences, Nahimovskiy prospect, 36, Moscow 117997, Russia; 2 Institute of Geology of Ore Deposits, Petrography, Mineralogy, and Geochemistry, Russian Academy of Sciences, Staromonetniy per. 35, Moscow 119017, Russia; 3 University of Johannesburg, Auckland Park Kingsway Campus, P.O. Box 524, Johannesburg, Republic of South Africa; 4 Institute of Environmental Problems of the North, Russian Academy of Sciences, nab. Severnoy Dviny, 23, Arkhangelsk 163000, Russia; 5 Norwegian Institute for Water Research, Gaustadalleen 21, Oslo No-0349, Norway

**Keywords:** Black Sea, Hydrogen sulfide, Sulfate, Sulfur isotopes, Oxygen isotopes, Hydrogen isotopes, Bottom Convective Layer, Sulfate reduction

## Abstract

The Black Sea is the largest euxinic basin on the Earth. The anoxic zone consists of the upper part water mass stratified by density, and the lower water mass homogenized relative to density (depth >1750 m), named the Bottom Convective Layer. To assess homogeneity and possible exchange of matter across the upper and lower boundaries of the Bottom Convective Layer, new data on stable isotope composition of S, O and H were obtained. Samples were collected in August 2008 and March 2009 from two stations located in the eastern central part of the Black Sea.

Distribution of δ^18^O and δD values of water for the entire water column did not vary seasonally. Appreciable differences were marked for δD value variation in the picnocline area (water depth 200-400 m) and in the BCL 5 m above the bottom that might be caused by penetration of intrusions with elevated portion of shelf modified Mediterranean Water. Observed linear relationship between δ^18^O (or δD) and salinity indicates that mixing water and salt occurs at the same time, and the deep water of the Black Sea has two end members: the high-salinity Mediterranean seawater and freshwater input.

In the Bottom Convective Layer, the average δ^34^S (H_2_S) was -40.6 ± 0.5‰ and did not vary seasonally. At the bottom (depth > 2000 m), ^34^S depletion down to –41.0‰ was observed. Our δ^34^S (SO_4_) data are by 2-3‰ higher than those measured previously for the Bottom Convective Layer. Sulfate from the aerobic zone with δ^34^S (SO_4_) = +21‰ corresponds to ocean water sulfate and that has not been subjected to sulfate reduction. Average δ^34^S (SO_4_) values for depths > 1250 m were found to be +23.0 ± 0.2‰ (1σ). Sulfur isotope composition of sulfate does not change in the Bottom Convective Layer and on its upper and lower boundaries, and does not depend on the season of observation.

## Background

The Black Sea is an enclosed inland sea with predominantly freshwater input at the surface. The only source of salt to the Black Sea is the Lower Bosporus Current (LBC) with salinity ~37% [1-3]. The annual outflow of water from the Black Sea through the Upper Bosporus Current is nearly more than twice the volume of inflowing LBC water [4,5]. The Black Sea water column is stratified by temperature and salinity down to the depth of ~1750 m. From this depth to the bottom, there is a Bottom Convective Layer (BCL), that makes 10.8% of the total volume of the Black Sea and is characterized by homogeneous distribution of potential temperature, salinity, alkalinity, hydrogen sulfide, ammonia and other parameters [1,6,7]. Homogeneity of physical and chemical characteristics is a result of convective mixing, driven by the geothermal flux from the underlying sediments [1]. This destabilizes the density stratification of the bottom waters. The vertical homogenization of the BCL occurs within a period of about 40 years [8]. The upward flux of heat and salt from the BCL contributes to the density stratification in the Black Sea water column over the BCL and formation of the main pycnocline. Another factor influencing the formation of the main pycnocline is winter mixing which supplies cold, low salinity oxygen rich water from the surface layer. A layer called the Cold Intermediate Layer (CIL) is formed above the main pycnocline. Below the main pycnocline, oxygen is rapidly depleted and hydrogen sulfide appears in the water at depths with potential density of 16.10–16.20 kg m^-3^[9]. Hydrogen sulfide concentration increases with depth maximum concentrations of 376 ± 4 μM in the BCL [7].

Earlier studies of the Black Sea water column have shown that isotope compositions of oxygen and hydrogen are determined by mixing the inflow of high salinity waters from the Sea of Marmara having characteristic isotopic signatures of δ^18^O = 1.58‰ and δD = 10.26‰ [10] with freshwater input, which represents the amount of river runoff and precipitation modified by evaporation. The lowest values of δ^18^O (−2.84‰) and δD (−23.03‰) were found at the surface layer (0–20 m) [10,11]. Within the analytical precision, the surface waters of the Black Sea are homogeneous in oxygen and hydrogen isotope compositions down to the CIL. This surface isotope composition is also typical for the Black Sea outflow through the Bosporus. The deep waters (depth over 500 m) are enriched in deuterium and ^18^O isotopes relative to the surface layer (δ^18^O = −1.77‰, δD = −15.87‰). The reason for this enrichment is mixing of surface water with the LBC inflow. Within the pycnocline, there is a linear relationship between oxygen and hydrogen isotope composition and potential density [8]. The published data on ^18^O and D isotope distribution for the Black Sea water are mainly related to the water masses shallower than 1500 m [8,12]. Oxygen and hydrogen isotope compositions of water in the BCL have not been studied in detail. For previous data see Swart [11].

The main source of hydrogen sulfide in the water column of the Black Sea is microbial reduction of sulfate [4,12,13]. As a result of dissimilatory sulfate reduction, sulfur in hydrogen sulfide becomes enriched in the light isotope, ^32^S. The value of δ^34^S (H_2_S) varies systematically over a range between −32.6 and −42.0‰ throughout the water column and, on the average, is −39.6 ± 1.3‰ [14]. There is little data for the sulfur isotope composition of hydrogen sulfide in the deep waters of the Black Sea (>1500 m). At these depths, Neretin et al. [14] observed slight δ^34^S enrichment up to −37.5‰. They proposed that the presence of ^34^S-enriched hydrogen sulfide in deep waters was due to addition of hydrogen sulfide by diffusion from sedimentary pore waters or by high sulfate reduction rates (SRR) in the uppermost “fluffy” layer [15].

There are significantly less data for the sulfur isotope composition of sulfate in the Black Sea water column than for dissolved hydrogen sulfide. The first results for sulfur isotope composition of sulfate in the Black Sea water to the depth of 2000 m were obtained at two stations by Vinogradov et al. [16]. The values of δ^34^S (SO_4_) varied over a small range of +18.6 to +19.5‰ relative to CDT (Canyon Diablo Troilite) and were close to the sulfur isotopic composition of sulfate for the Bosporus Strait waters +19.8‰. Later, Sweeney and Kaplan [12] found that ^32^S enrichment of hydrogen sulfide in the Black Sea resulted in an increase in the sulfur isotopic composition of sulfate from +18.2 to +20.2‰ with increasing depth. Along with the change of sulfur isotope composition, sulfate content increases (because salinity increased) from 16.2 mM at 125 m to 18.1 mM at 1000–1400 m. Only one sample from the depth of 500 m (δ^34^S (SO_4_) = +19.5‰) was analyzed by Fry et al. [13]. Distribution of δ^34^S (SO_4_) values with depth from surface to 180 m was presented by Neretin et al. [17]. In the aerobic zone down to the depth of 100 m, sulfur isotope composition in sulfate was nearly constant 20.5-20.7‰ relatively VCDT. Enrichment of ^34^S in sulfate up to +20.8‰ was coincided with maximum of sulfate-chlorine ratio and was obtained from 20–30 m above H_2_S appearance. Since the work of Neretin et al. [17] there have been no new measurements of sulfur isotope composition of sulfate in the water column of the Black Sea, especially in the BCL.

In this paper we present new data on the isotopic composition of oxygen and hydrogen for the entire water column of the Black Sea. The main objective of this presentation is to consider the variability in the isotope composition of oxygen and hydrogen in BCL to determine the homogeneity of their distribution. Based on the calculated isotopic characteristics for fresh water input and the water balance of the Black Sea [5], we can obtain the average annual isotope parameters for water vapor.

**Figure 1:**
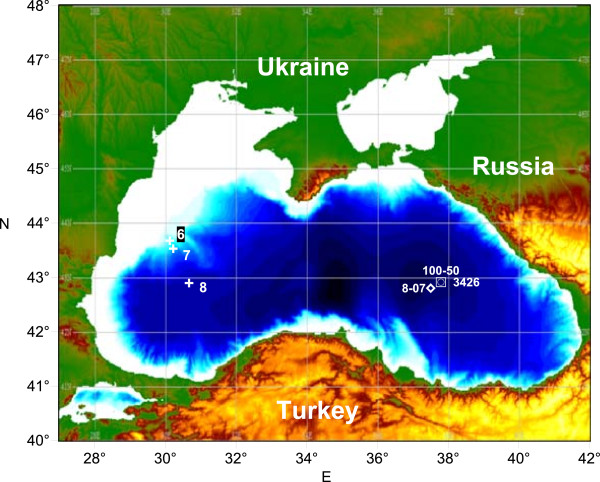
**Stations sampled during cruises: R/V *Akvanavt *** **2008 (station 3426 at 42°54′39″N, 37°47′44″E, water depth 2141 m) and R/V *Professor Shtokman *** **2009 (station 100–50 at 42°54′43″N, 37°47′36″E, water depth 2142 m). Also it is shown station 8–07 [**[20]**], and stations 6, 7 and 8 [**[14]**,**[21]**].**

Convective mixing in the BCL should tend to produce homogeneous sulfur isotope compositions of hydrogen sulfide and sulfate. However, their composition may vary on the upper and lower boundaries of the BCL. The reason for the possible ^32^S depletion in sulfur of H_2_S at the lower boundaries of the BCL can be the increase of sulfate reduction rate in surface sediments resulting from seasonal increase in supply of particulate organic matter [15,18,19] or from increasing influence of hydrogen sulfide flux from sediments with decreasing the distance to the bottom [14,20]. Particular attention was given to studying sulfur isotope composition of hydrogen sulfide at the upper boundaries of the BCL and overlying water column up to the depth 1250 m. From two samplings at one station located in the eastern central part of the Black Sea (Figure 1), we examined possible temporal changes in distribution of stable isotopes of oxygen and hydrogen in water, and sulfur isotope composition of hydrogen sulfide and sulfate.

Based on the data on sulfur isotopic composition in sulfate in the BCL and suggestion on restricted mass exchange between BCL and overlying part of the sea and sediments, we could calculate mass fraction of sulfate lost during sulfate reduction and compare this finding with the sulfate-chlorine ratio in BCL from early investigations [4,19,22,23].

## Materials and methods

### Sampling sites

The water column of the Black Sea was sampled during cruise 146 of R/V *Akvanavt* (Station 3426, August 2008) and cruise 100 of R/V *Professor Shtokman* (Station 100–50, March 2009) (see Figure 1). Water samples were collected using twelve 5-L Niskin bottles mounted on a Seabird rosette system equipped with a SBE 19 CTD, WetLab ECO-AFL profiling fluorometer for chlorophyll-a, WetLab c-beam transmissometer and altimeter. During cruise 100 of R/V *Professor Shtokman,* the Niskin bottles were slightly pressurized with Ar during sample withdrawal to minimize O_2_ contamination [24]. Hydrogen sulfide concentrations (below 30 μM) were determined by the methylene blue method [25], while higher concentrations were analyzed by iodometric (volumetric) titration. During cruise 100 of R/V *Professor Shtokman,* both methods were used to determine hydrogen sulfide content in the anoxic zone [24]. All hydrogen sulfide determinations and sample fixation for isotope analyses were performed onboard ship immediately after sample retrieval.

### Determining of the oxygen and hydrogen isotope composition of water

Determination of oxygen isotope composition was performed by isotope equilibration with CO_2_. Measurements were carried out in a continuous flow of helium (CF IRMS) by a DELTA V + mass spectrometer, together with GasBench II and autosampler PAL peripheral devices. The sample volume was 0.5 ml of water. Reference samples of IAEA OH-1, OH-2, OH-3 and OH-4 were used as reference standards, absolute values of which were calibrated on the VSMOW scale. To correct for possible instrumental drift during the analysis of each series consisting of 40 samples, the internal laboratory standard was measured every 6–8 samples. Reproducibility of δ^18^O values was ± 0.2‰ (1σ). Hydrogen isotope analysis was carried out using high-temperature reduction of hydrogen from water on Cr^0^. The sample volume was 1 μL of water. Reference samples of IAEA OH-1, OH-2, OH-3 and OH-4 were used as standards, absolute values of which were calibrated on the VSMOW scale. The measurements were performed in a dual-inlet mode using a DeltaPlus mass spectrometer with a peripheral H/Device. Reproducibility of δD values was ± 0.3‰ (1σ). δ^18^O and δD values are expressed relative to VSMOW:

$$\delta D_{\mathrm{sample}}={\left(D/H\right)}_{\mathrm{sample}}/{\left(D/H\right)}_{\mathrm{VSMOW}}–1\;\mathrm{and}$$

$$\delta^{18}O_{\mathrm{sample}}={\left({}^{18}O{/}^{16}O\right)}_{\mathrm{sample}}/{\left({}^{18}O{/}^{16}O\right)}_{\mathrm{VSMOW}}–1.$$

### Method of preparation of the seawater samples for sulfur isotope analysis

50 ml of zinc acetate solution (50 g zinc acetate, 10 g sodium acetate and 0.5 g sodium chloride in 1 L distilled water) was poured into a polypropylene container (volume 1 L) prior to sampling, then seawater from the Niskin bottle was added to make 1 L of final solution. After intensive mixing for one minute and settling during one hour, the solution with ZnS precipitate was filtered using 0.45 μm HA Millipore filter. The filter was then air dried.

After filtration of about one half of the ZnS solution, a portion of the filtrate (150 ml) was collected in a glass beaker, and 1–2 ml of 6 M HCl was added. The beaker with the filtrate was heated to boiling at constant stirring, and then 20 ml of a 10% BaCl_2_ solution was added. After cooling, the BaSO_4_ precipitate was filtered with a 0.45 μm Millipore filter. The filter was washed with distilled water and 0.05 ml of 6 M HCl, and then the filter was air dried. To determine the sulfur isotope composition of sulfate in seawater in the aerobic zone, the sulfate precipitation from surface water (depth 1.2-1.6 m) was carried out only after the step of adding the BaCl_2_ solution as described above.

To transfer ZnS to Ag_2_S, the filter with ZnS precipitate was placed in a flask for hydrogen sulfide distillation. The filter was first acidified with 20 ml 6 M HCl under Ar, and the released sulfide was quantitatively precipitated in a trap containing 100 ml of aqueous silver nitrate (0.5% w/v). After purging with argon for 5 minutes, 80 ml distilled water was added into the flask. Then the reaction flask was heated to boiling. After Ag_2_S coagulation on the hot plate, it was cooled and settled for 12 hours and then filtered with 0.45 μm Millipore filter. The filter was washed with 5% NH_4_OH and dried.

### Sulfur isotope analyses

Conversion of sulfur to SO_2_ was conducted in high temperature reactor filled with Cu^0^ and WO_3_ using elemental analyzer FlashEA HT 1112. The sulfur isotope composition in SO_2_ gas was measured in a continuous flow of helium using CF-IRMS method by a DELTA V + mass spectrometer (Finnigan, Germany). During the measurements, ion currents corresponding to the masses 64 and 66 were detected. Weight of the sample for measurements of sulfur isotope composition was 400 μg as Ag_2_S, and 360 μg as BaSO_4_. Prior to analyses, V_2_O_5_ was added to the capsule with BaSO_4_ in the mass ratio of 1:1. Samples and standards in tin capsule were placed in the cells of a 32 position autosampler. International reference standards for Ag_2_S (IAEA-S-1, IAEA-S-2 and IAEA-S-3) and BaSO_4_ (NBS 127 and IAEA-SO-5) were measured at the beginning and at the end of each series. All data are reported relative to VCDT with accepted reference sample compositions: IAEA-S-1 (−0.3‰), IAEA-S-2 (+22.67‰), IAEA-S-3 (−32.55‰), IAEA-SO-5 (+0.49‰) and NBS 127 (+21.1‰). In this work the δ^34^S values of −0.30 ± 0.15‰ (n = 40), +22.55 ± 0.14‰ (n = 11), −32.51 ± 0.18‰ (n = 23) and +21.14 ± 0.14‰ (n = 23) were obtained for reference samples IAEA-S-1, IAEA-S-2, IAEA-S-3 and NBS 127 respectively.

For analysis of sulfur in the form of Ag_2_S, the basic standard was silver sulfide IAEA-S-3, the isotope composition of which is the closest to the composition of sulfur sulfide in the water of the Black Sea (δ^34^S_VCDT_ = −32.55‰). Calibration of the working standard and calculation of stretching factor were performed daily by measuring three international reference standards IAEA-S-1, IAEA-S-2 and IAEA-S-3. The drift of the instrument was corrected by measuring the IAEA-S-3 standard after every 6 samples. Reproducibility of replicate determinations was better than ± 0.2‰.

NBS 127 standard (seawater sulfate) was used as the standard for analyzing sulfate isotope composition. The oxygen isotope composition of this standard is very close to that of seawater sulfate. Thus, after our analyses an additional correction of δ^34^S value due to the oxygen isotope composition was not required. Correction for instrument drift was based on the measurements of the NBS 127 standard. Reproducibility of the method was better than ± 0.2‰. Sulfur isotope results are presented relative to Vienna Canyon Diablo Troilite (VCDT) using standard δ notation [26]:

$$\delta^{34}S_{\mathrm{sample}}={\left({}^{34}S{/}^{32}S\right)}_{\mathrm{sample}}/{\left({}^{34}S{/}^{32}S\right)}_{\mathrm{VCDT}}–1$$

Fractionation of sulfate-sulfide was calculated by:

$$\begin{array}{l} \epsilon =\alpha –1,\mathrm{where}\;\alpha \;\text{is fractionation}\;\mathrm{factor},\\ \alpha =\left(\delta^{34}S\left({\mathrm{SO}}_{4}\right)+1\right)/\left(\delta^{34}S\left(H_{2}S\right)+1\right). \end{array}$$

## Results

### Thermohaline properties of the Bottom Convective Layer

**Figure 2:**
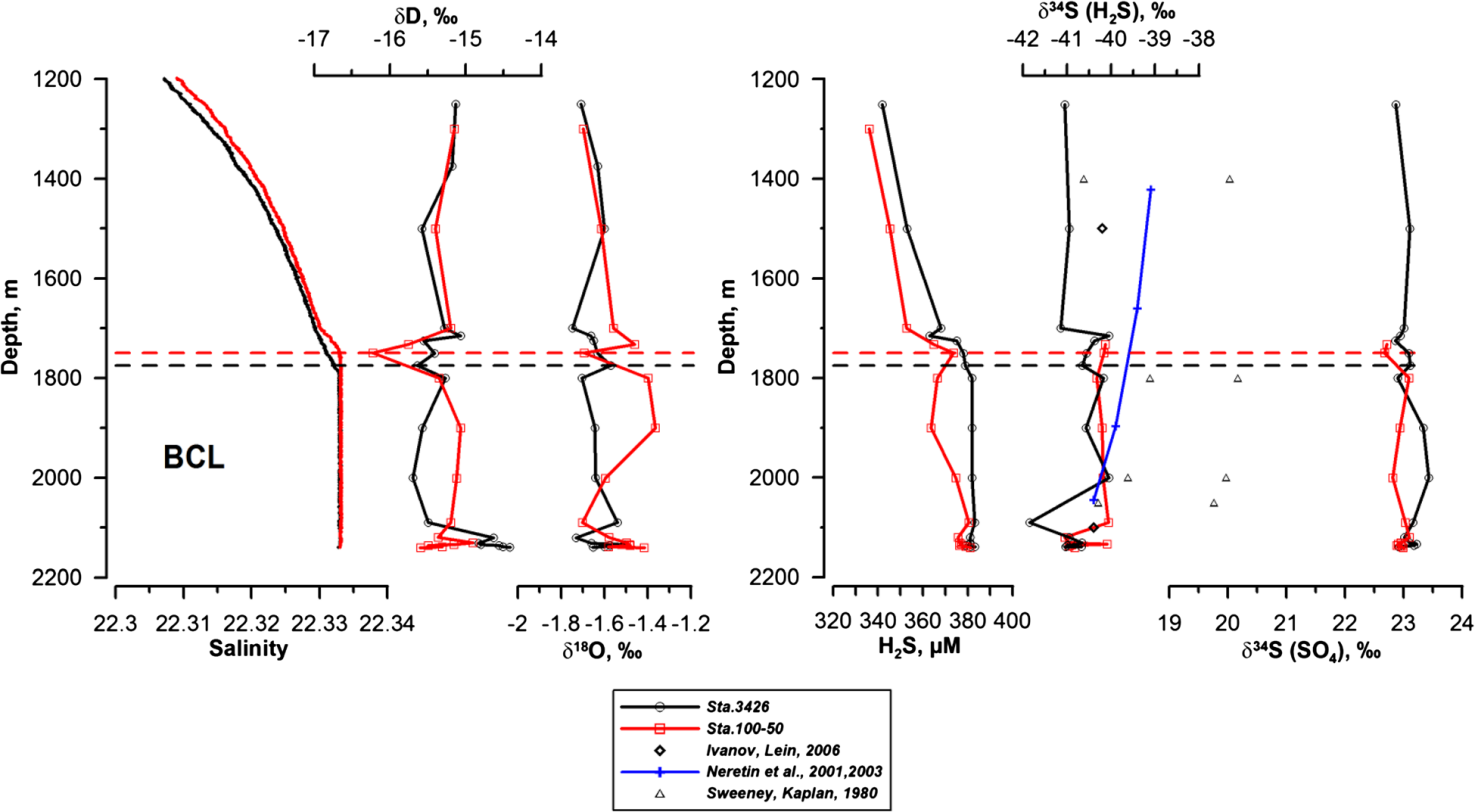
**The vertical distribution of salinity, δ**^**18**^**O, δD (left panel) and H**_**2**_**S content, δ**^**34**^**S (H**_**2**_**S) and δ**^**34**^**S (SO**_**4**_**) (right panel) in the lower part (>1200 m) of the stations 3426 and 100–50 water column.** The dashed lines show the upper boundary of the BCL. The data on δ^34^S (H_2_S) value from [28], [14,21] and [12] are also presented. The lowest δ^34^S (H_2_S) value from [21] is shown for sulfur isotope composition of hydrogen sulfide from pore water (10–18 cm depth of sediments at station 8).

The bottom layer of the Black Sea water column (thickness 400–500 m) is characterized by constant vertical distributions of potential temperature (θ = 8.886 – 8.896°C), salinity (S = 22.321 – 22.337) and potential density (σ_θ_ = 17.223 – 17.236 kg m^-3^), as well as by neutral or weak negative stability, which indicates the presence of convective processes at the bottom [1,8,27]. The values of potential temperature, salinity and density in the BCL have varied slightly according to different authors. These variations might relate both to the accuracy of measurements and horizontal and temporal variability of the BCL. Averaged data for BCL parameters obtained during the cruise of the R/V *Akvanavt* in the summer 2008 and of the R/V *Professor Shtokman* in the early spring 2009 are θ = 8.900°C, S = 22.333, σ_θ_ = 17.233. At the upper boundary of the Bottom Convective Layer, abrupt changes of temperature, salinity and potential density gradients were observed (Figure 2).

### Oxygen and hydrogen isotope composition of seawater

**Figure 3:**
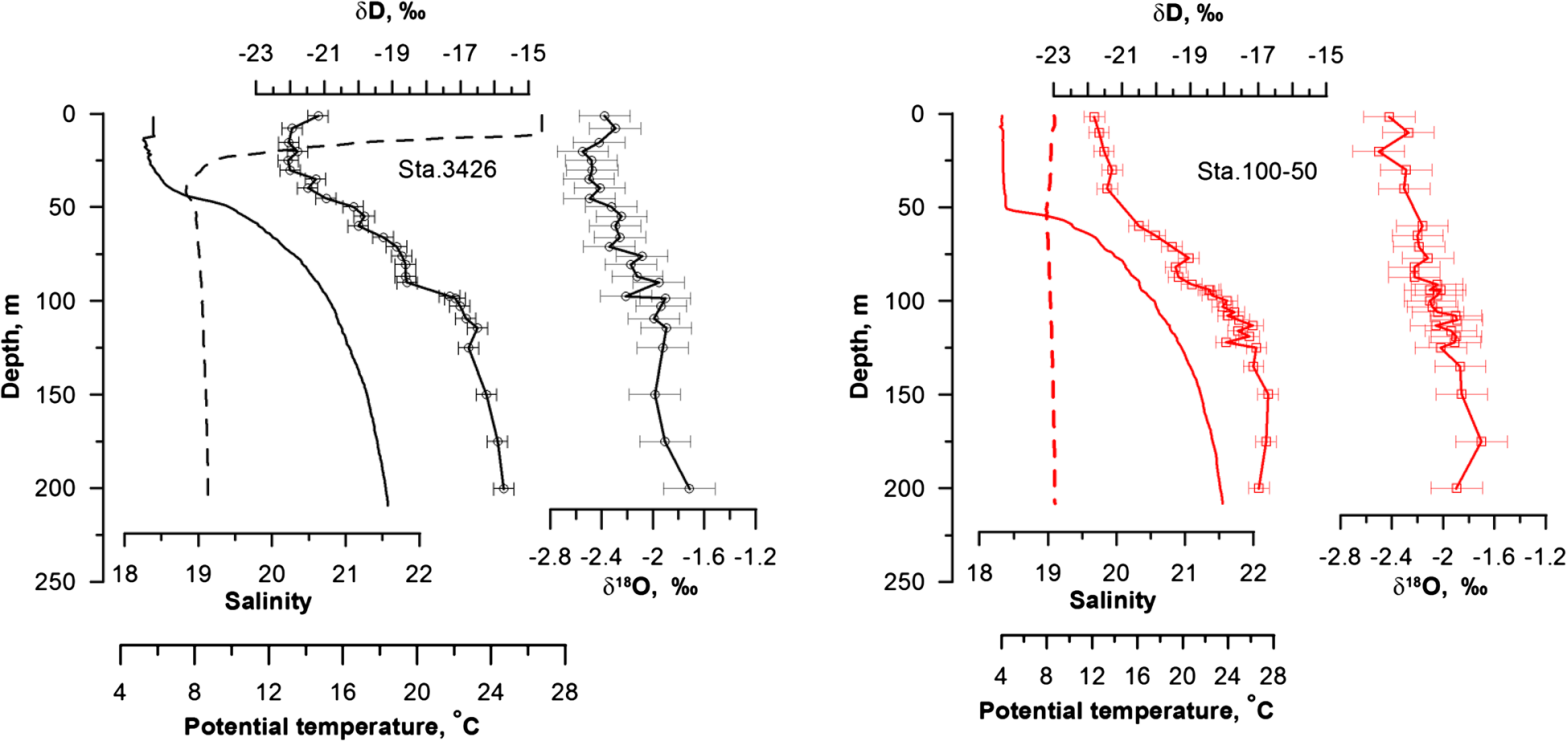
**The vertical distribution of salinity, potential temperature, δ**^**18**^**O and δD in the upper part (<200 m) of the stations 3426 and 100–50 water column. Error bars (1σ) for δ**^**18**^**O and δD values are shown.**

Results of δ^18^O and δD value determination are shown in Tables 1 and 2. Distribution of temperature, salinity, δ^18^O and δD with depth of the water column at stations 3426 and 100–50 are shown in Figures 2 and 3. It can be seen that hydrogen and oxygen isotope compositions are enriched with heavy isotopes with increasing water depth. The values of δ^18^O are decreased from −1.4‰ in the BCL to −2.5‰ in the surface water. Hydrogen isotope composition varies to a greater extent: from −22.1‰ in the surface layer to −14.4‰ in the BCL.

**Table 1:** Salinity, oxygen and hydrogen isotope composition of water column at station 3426

**Depth, m**	**Salinity**	**δ**^**18**^**O, ‰**	**δD, ‰**
1.2	18.398	−2.4	−21.2
7.9	18.408	−2.3	−21.9
15.6	18.274	−2.4	−22.0
20.2	18.298	−2.5	−21.8
25.1	18.395	−2.5	−22.1
30.3	18.411	−2.5	−22.0
34.9	18.464	−2.5	−21.2
39.8	18.544	−2.4	−21.5
45.4	19.000	−2.5	−20.9
49.9	19.167	−2.3	−20.1
55.0	19.518	−2.2	−19.8
60.1	19.794	−2.3	−20.0
66.0	19.992	−2.3	−19.3
71.0	20.117	−2.3	−18.9
76.1	20.243	−2.1	−18.7
80.6	20.382	−2.2	−18.6
87.0	20.529	−2.1	−18.6
90.2	20.627	−2.0	−18.6
97.4	20.766	−2.2	−17.3
98.7	20.782	−1.9	−17.2
103	20.848	−1.9	−17.0
109	20.917	−2.0	−16.8
115	20.986	−1.9	−16.5
125	21.108	−1.9	−16.8
150	21.300	−2.0	−16.2
175	21.421	−1.9	−15.9
200	21.532	−1.7	−15.7
250	21.698	−1.7	−16.6
300	21.818	−1.8	−15.1
350	21.897	−1.7	−15.2
400	21.962	−1.7	−15.2
450	22.016	−1.7	−15.1
500	22.061	−1.7	−15.8
550	22.103	−1.7	−15.4
600	22.133	−1.8	−15.6
650	22.166	−1.8	−15.3
700	22.193	−1.9	−15.5
750	22.213	−1.7	−15.6
801	22.234	−1.8	−15.0
900	22.264	−1.7	−15.0
1000	22.283	−1.7	−15.3
1125	22.302	−1.8	−15.1
1250	22.313	−1.7	−15.1
1375	22.321	−1.6	−15.2
1500	22.325	−1.6	−15.6
1700	22.331	−1.7	−15.3
1715	22.331	−1.7	−15.1
1725	22.331	−1.7	−15.6
1751	22.332	−1.6	−15.4
1775*	22.333	−1.6	−15.6
1801*	22.333	−1.7	−15.3
1900*	22.333	−1.6	−15.6
2000*	22.333	−1.6	−15.7
2090*	22.333	−1.5	−15.5
2120*	22.333	−1.7	−14.6
2131*	22.333	−1.7	−14.8
2134*	22.333	−1.5	−14.8
2137*	22.333	−1.6	−14.6
2138.7*	22.333	−1.6	−14.5
2139.4*	22.333	−1.7	−14.4

(*) – BCL samples.

**Table 2:** Salinity, oxygen and hydrogen isotope composition of water column at station 100-50

**Depth, m**	**Salinity**	**δ**^**18**^**O, ‰**	**δD, ‰**
1.6	18.290	−2.4	−21.8
10.0	18.294	−2.3	−21.7
20.1	18.291	−2.5	−21.5
30.0	18.291	−2.3	−21.3
40.1	18.292	−2.3	−21.4
59.9	18.942	−2.2	−20.5
65.0	19.362	−2.2	−20.0
71.0	19.673	−2.2	−19.5
77.0	19.992	−2.1	−19.0
81.9	20.135	−2.2	−19.4
87.1	20.322	−2.2	−19.3
91.1	20.460	−2.0	−19.0
94.0	20.474	−2.1	−18.4
94.0	20.474	−2.0	−18.5
96.9	20.565	−2.0	−18.4
100	20.616	−2.1	−17.9
103	20.673	−2.1	−18.1
106	20.736	−2.0	−17.7
108	20.786	−1.9	−17.9
110	20.834	−1.9	−17.6
113	20.885	−2.1	−17.2
116	20.936	−1.9	−17.6
119	20.971	−1.9	−17.3
122	21.009	−1.9	−17.9
125	21.050	−2.0	−17.1
135	21.143	−1.9	−17.1
150	21.273	−1.9	−16.7
175	21.424	−1.7	−16.8
200	21.516	−1.9	−17.0
300	21.794	−1.7	−16.3
400	21.943	−1.6	−16.2
700	22.187	−1.6	−14.9
1000	22.285	−1.6	−14.9
1300	22.316	−1.7	−15.1
1500	22.324	−1.6	−15.4
1700	22.330	−1.6	−15.2
1732	22.332	−1.5	−15.8
1749*	22.333	−1.7	−16.2
1800*	22.333	−1.4	−15.4
1900*	22.333	−1.4	−15.1
2001*	22.333	−1.6	−15.1
2090*	22.333	−1.7	−15.2
2120*	22.333	−1.6	−15.4
2131*	22.333	−1.5	−14.9
2134*	22.333	−1.5	−15.2
2136.5*	22.333	−1.5	−15.5
2138.5*	22.333	−1.6	−15.3
2140.6*	22.333	−1.4	−15.6

(*) – BCL samples.

**Figure 4:**
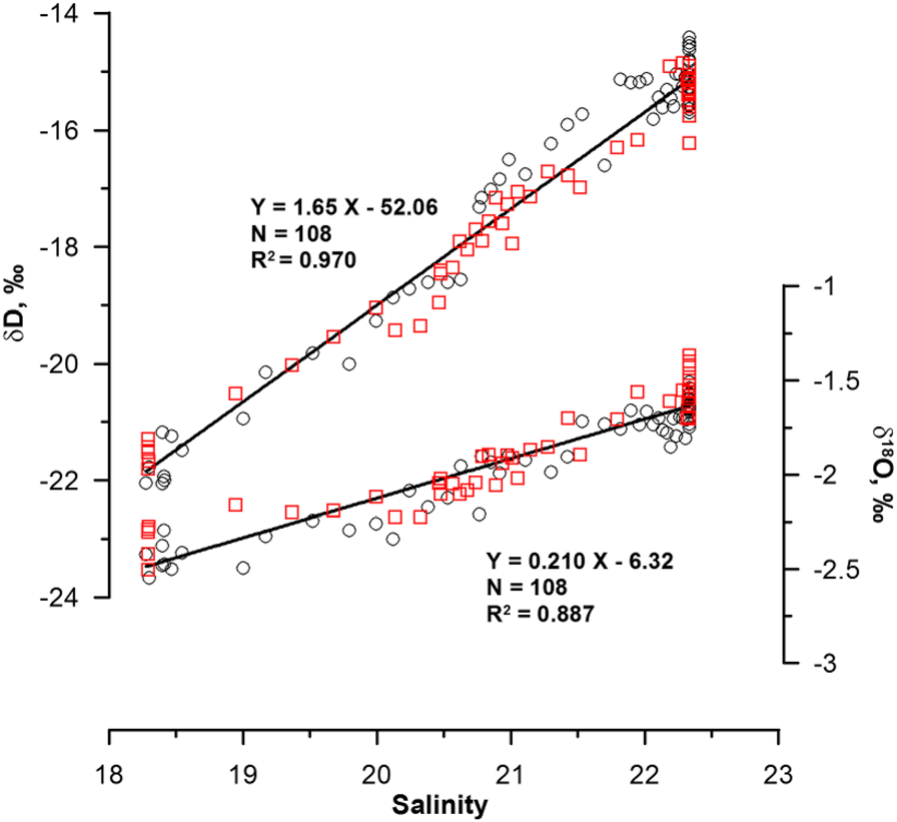
**δ**^**18**^**O and δD versus salinity plot for the Black Sea water column at stations 3426 (black) and 100–50 (red).**

There are linear relationships between the hydrogen and oxygen isotope distributions and salinity for both stations (Figure 4). This dependence could result from mixing of two solutions with different amount of salt and isotope compositions, such as in the estuaries. The only source of salt in the Black Sea is the inflow of high salinity LBC water. According to Latif et al. [2], the salinity of the LBC is close to 37‰, and the hydrogen and oxygen isotope composition of this water is δD = +10.3‰ and δ^18^O = +1.58‰ respectively [10]. The Black Sea is a basin dominated by freshwater input at the surface [5]. Based on data for the Danube River [29,30], the river discharge differs significantly from the LBC water in salt, oxygen and hydrogen isotope compositions. According to Rank et al. [30], water of the lower Danube River (sampling in September), has average oxygen and hydrogen isotope compositions of δ^18^O = −9.73 ± 0.06‰ and δD = − 69.44 ± 0.81‰, respectively.

Water masses in the water column of the Black Sea can be distinguished according to their salinity and isotope composition. The structure of the upper layer of the Sea is determined by convective mixing in the winter [31] between the surface and the core of the CIL (at a density of σ_θ_ = 14.5, which varies from a depth of 39.8 m at station 3426 and 59.9 m at station 100–50). Salinity of this upper layer varies from 18.3 to 18.9 at both stations and does not differ significantly in winter and summer despite the presence of a negative thermocline in the summer 2008 (see Figure 3). δ^18^O values vary from −2.2 to −2.5‰, apparently not depending on the season. In the Bosporus and in the coastal zone within the area of river inflow, the oxygen isotope composition of surface water can be as low as −2.8‰ [10]. δD value varies from −22.1 to −21.8‰ in the surface layer (see Tables 1 and 2). The oxygen and hydrogen isotope compositions of water, like salinity, undergo the greatest change in the main pycnocline between the core of the CIL and the depth of 500 m. Below 500 m and down to the bottom, δ^18^O and δD values change little.

**Figure 5:**
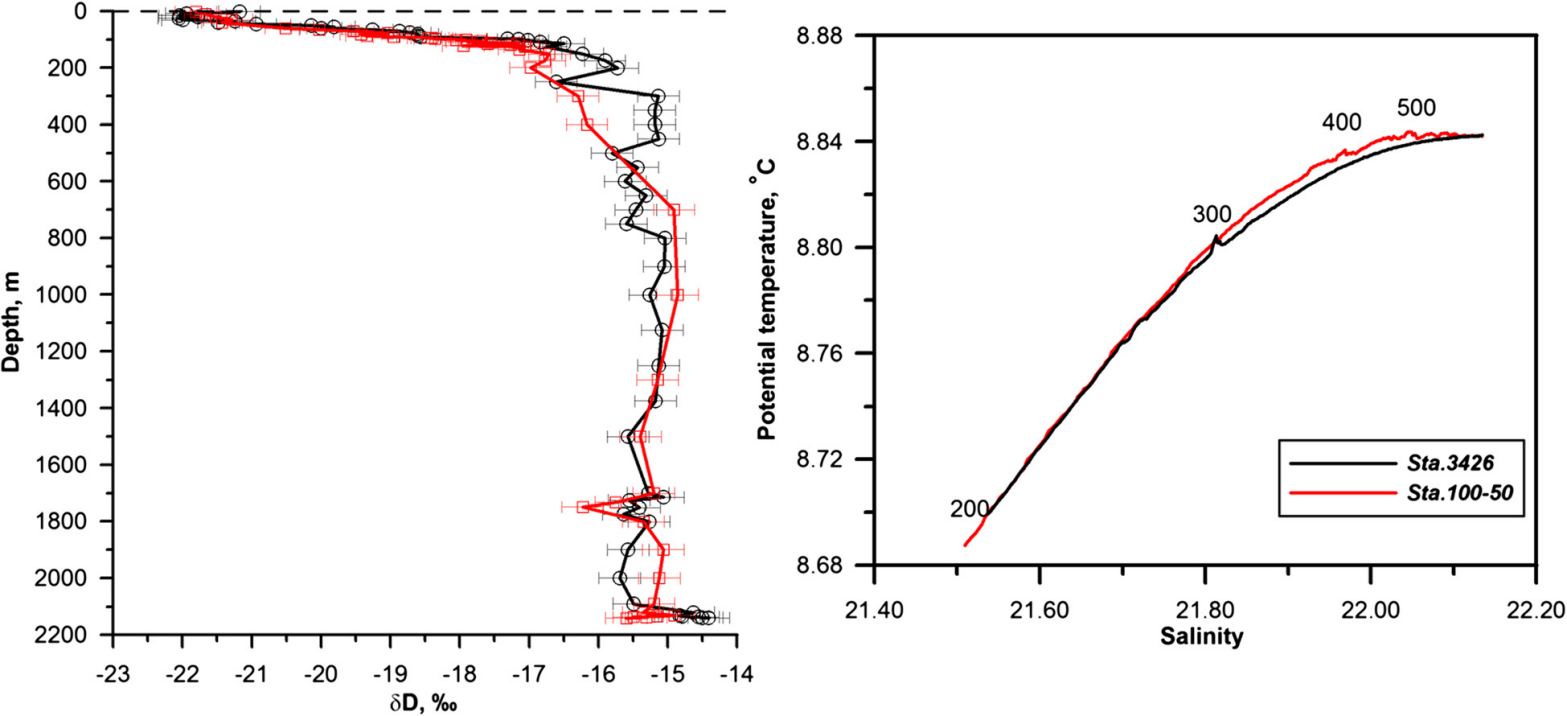
**The vertical distribution of δD values versus depth of the stations 3426 (black) and 100–50 (red) water column (left panel).** Potential temperature – salinity diagram for 200 – 600 m depth of water column at station 3426 (black) and station 100–50 (red) (right panel). Error bars for δD values correspond to 1σ

Hydrogen isotope composition is a more sensitive parameter of water sources than the oxygen isotope composition. δD value variations in the Black Sea water reach 8‰ at accuracy of determination 0.3‰. Comparison of δD distribution in the main pycnocline between two stations indicates that at depths of 175, 200, 300 and 400 m the differences in the hydrogen isotope composition are equal to or exceed 3σ (from 0.9 to 1.2‰) (Figure 5). At station 3426 in summer 2008 the isotopic composition of hydrogen was greater at these depths and the potential temperature was lower than in spring 2009 at station 100–50. Similar differences in the δD values were met in bottom waters at depths greater than 2137 m (from 0.8 to 1.2‰). Significant differences in salinity or temperature in the bottom layer were not detected (Figures 2 and 5).

### Sulfur isotope composition of hydrogen sulfide and its variation

Sulfur isotope composition of dissolved sulfide was analyzed at station 3426 at depths 1250–2139.4 m (summer 2008) and at station 100–50 at depths 1732–2140.6 m (spring 2009) (Tables 3 and 4). In summer 2008, isotope composition remained constant (from −41.0 to −41.2‰) down to 1700 m (see Figure 2). At the upper boundary of BCL, there was an increase in δ^34^S (H_2_S) up to −40‰. In the BCL, the average δ^34^S (H_2_S) was found to be −40.8‰ (SD = 0.5‰, RSD = 1.2%) with a range from −40.0 to −41.9‰ for 11 samples. Down to the bottom (less than 52 m from the bottom), there was slight depletion in δ^34^S (H_2_S) from −40.3 to −41.0‰.

**Table 3:** **H**_**2**_**S content and sulfur isotope composition of hydrogen sulfide and sulfate in seawater of station 3426**

**Depth, m**	**H**_**2**_**S, μM**	**δ**^**34**^**S (H**_**2**_**S), ‰**	**δ**^**34**^**S (SO**_**4**_**), ‰**
1.2	−	−	21.0
1250	342	−41.1	22.9
1500	353	−41.0	23.1
1700	368	−41.2	23.0
1715	363	−40.0	22.9
1725	375	−40.4	22.9
1751	378	−40.6	23.1
1775	379	−40.7	23.1
1801	382	−40.2	22.9
1900	382	−40.6	23.3
2000	382	−40.0	23.4
2090	383	−41.9	23.2
2120	381	−40.9	23.0
2131	381	−40.7	23.1
2134	381	−40.8	23.2
2137	379	−41.0	23.2
2138.7	381	−40.7	22.9
2139.4	383	−41.0	22.9

“−”: no data.

Data obtained by iodometric titrations are presented for H_2_S content.

**Table 4:** **H**_**2**_**S content and sulfur isotope composition of hydrogen sulfide and sulfate in seawater of station 100-50**

**Depth, m**	**H**_**2**_**S, μM**	**δ**^**34**^**S (H**_**2**_**S), ‰**	**δ**^**34**^**S (SO**_**4**_**), ‰**
1.6	−	−	21.1
1732	365	−40.1	22.7
1749	374	−40.2	22.7
1800	367	−40.3	23.1
1900	364	−40.2	22.9
2001	375	−40.2	22.8
2090	380	−40.1	23.0
2120	376	−41.1	23.1
2131	376	−41.0	23.0
2134	377	−40.1	22.9
2136.5	376	−40.9	22.9
2138.5	379	−41.0	23.0
2140.6	381	−40.8	23.0

“−”: no data.

Data obtained by iodometric titrations are presented for H_2_S content.

The same trends were observed near the bottom during the spring survey of 2009. Below 2120 m, there was a depletion in δ^34^S (H_2_S) to −41‰ (except for one sample from the depth of 2134 m). Above 2120 m in the BCL, the value of δ^34^S (H_2_S) was essentially constant. The average δ^34^S (H_2_S) value for all 11 samples from BCL at station 100–50 was −40.5‰ (SD = 0.4%, RSD = 1.0‰) and did not differ significantly from that collected in the summer 2008. Depletions in the sulfur isotope composition by almost 1‰ in the near bottom area were accompanied by an increase in concentrations of hydrogen sulfide (see Figure 2).

### Sulfur isotope composition of sulfate

Within the Bottom Convective Layer at station 3426, the value of δ^34^S (SO_4_) did not change significantly (see Table 3 and Figure 2). The average value for 11 samples was +23.1 ± 0.2‰. At station 100–50 in spring 2009, the value of δ^34^S (SO_4_) averaged at +22.9 ± 0.1‰ for 11 samples (see Table 4). The average value for these two stations was equal to +23.0 ± 0.2‰. In the aerobic zone of the Black Sea at a depth of 1.2-1.6 m, the value of δ^34^S (SO_4_) was +21.0‰ at station 3426, and +21.1‰ at station 100–50 in respect of VCDT standard. These data do not differ from the sulfate standard of the ocean water NBS 127.

## Discussion

### Sources of waters determined from oxygen and hydrogen isotope composition

Distribution of oxygen and hydrogen isotope composition of water in the Black Sea water column is a result of mixing the LBC inflow and freshwater input, the components of which are the river runoff and meteoric precipitation. Specifically, all deep water below the CIL falls on roughly a linear relationship between two end members, the CIL and the Lower Bosporus inflow. When the linear relation deviates it is mostly due to variability in the signatures of the CIL end-member. Previously, hydrogen and oxygen isotope composition in the BCL was considered by Swart [11]. He showed that the Black Sea deep waters (including BCL) had essentially constant composition (δ^18^O = −1.65‰, δD = −14.29‰), while the surface layer (down to the core of CIL) was depleted in ^18^O and D. Rank et al. [10] obtained data for multiple stations, mainly in the western part of the Black Sea and studied the relationship between oxygen or hydrogen isotope composition of water and potential density. Below the CIL and down to about 1500 m (the maximum depth investigated), the relationship was linear. In our opinion, the linear dependence of hydrogen and oxygen isotope composition on potential density found by Rank et al. [10] and Özsoy et al. [8] is due to the linear relationship between δ^18^O and δD values and salinity, contribution of which to the calculation of potential density is predominant [1]. The dependence of δ^18^O and δD with salinity is linear over the entire salinity range except for the Bottom Convective Layer at depths more than 1750 m (see Figure 4). Salinity in the BCL is constant, while hydrogen (with the exception of data below 2137 m depth) and oxygen isotope compositions vary over a small range (see Tables 1 and 2), which is comparable with reproducibility of the determination method. This means that changes in the isotopic composition of hydrogen and oxygen in the BCL are not significant.

In the near bottom area of BCL and in the pycnocline at depth of 200–400 m the difference in δD value between two stations exceeded 3σ (see Figure 5). It was found that at the same salinity the temperature in pycnocline of station 3426 was lower than at the station 100–50. A possible explanation for these differences could be propagation of intrusions from the Bosporus area, previously observed in the eastern Black Sea [32]. At station 3426, a positive anomaly in the isotope composition of hydrogen was accompanied by low temperature. Negative temperature anomalies are widespread in the lenses and intrusions in the Bosporus area [3]. Positive δD anomaly in the pycnocline and in the bottom layer may indicate a larger proportion of shelf modified Mediterranean waters. Inflow of Mediterranean waters varies considerably over the year [33]. Possibly the increase of LBC inflow led not only to the formation of lenses in pycnocline but also to their appreciable penetration into the bottom part of the BCL. Probably, these findings require further observations.

**Figure 6:**
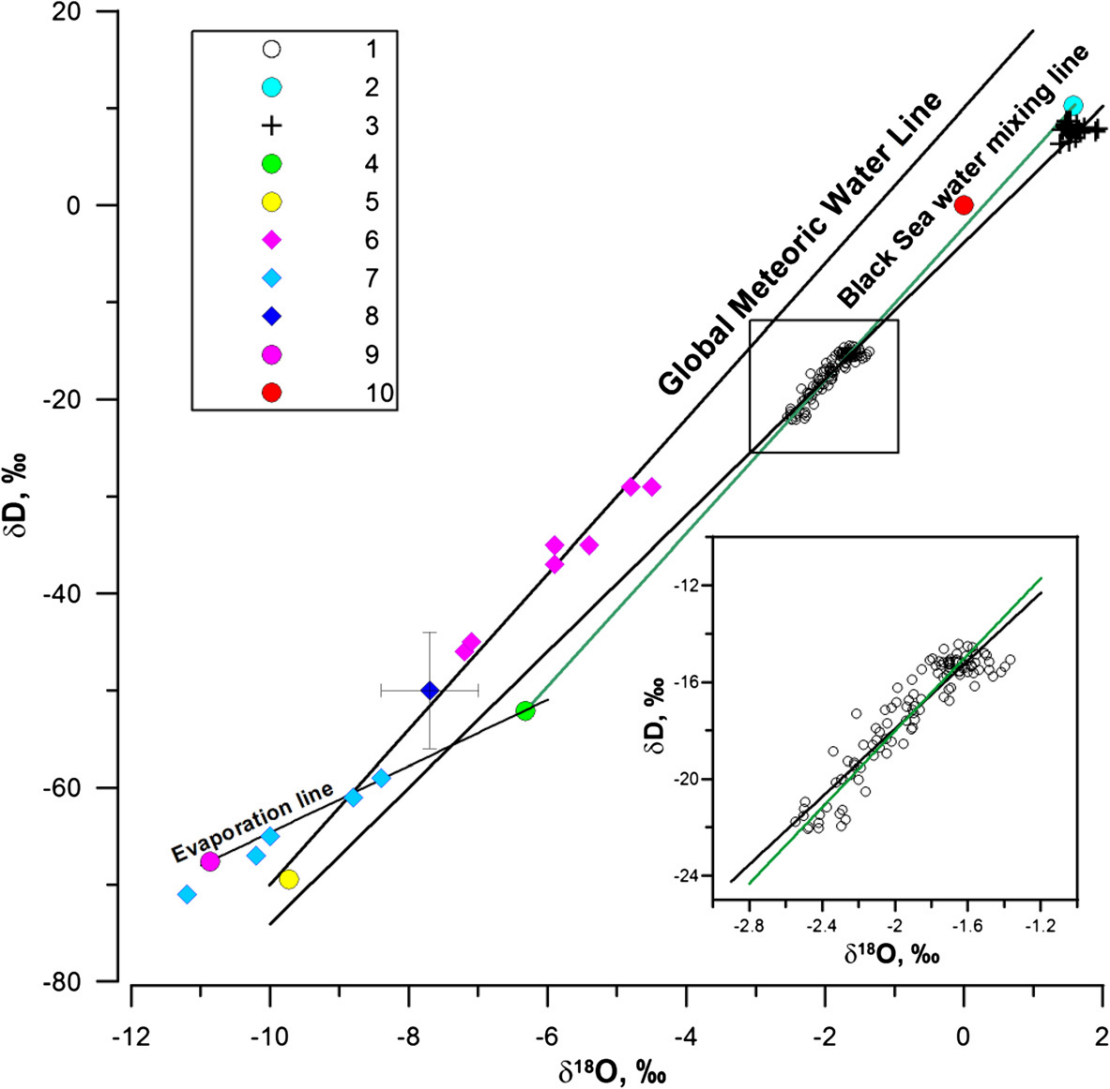
**δ**^**18**^**O versus δD values in the Black Sea water (1) and its possible sources.** The LBC composition is close to seawater of the Marmara Sea (depth >50 m) (2) [10] and the East Mediterranean Sea (3) [34]. Fresh water input (4) consists of river runoff (5) and precipitation (6–8) which are modified by evaporation (9). River runoff is presented by average isotope composition of the lower part Danube River (5) [30], data on annual average precipitation composition (6) are from [35], data on warm months (7) and cold months (8) are from [36]. Evaporation line is calculated from water mass balance in the Black Sea (see details in text). SMOW isotope composition (10) is also shown.

Generally, the data for hydrogen and oxygen isotope composition from the two stations do not differ, and observations can be considered as a single data array. The linear dependence of δ^18^O and δD on salinity shows that the Black Sea water composition has two possible sources of the water. With data for the LBC inflow δ^18^O = 1.58‰ and δD = 10.26‰ [10], from the equations of dependence S - δ^18^O and S - δD we get identical salinity of 37.7. This salinity is typical for that of the LBC water inflow, and that of the Marmara Sea and the Mediterranean Sea [2,3,34]. If salinity tends to 0, we get δ^18^O = −6.32‰ and δD = −52.1‰. These isotope parameters, apparently, are characteristic of the freshwater component, the isotope composition of which is influenced by three factors: the river runoff into the Black Sea basin, precipitation, and evaporation from the surface of the sea (Figure 6). By combining the linear equations of the δ^18^O and δD from salinity (see Figure 4)

$$\delta^{18}O=a_{1}\times S+b_{1}\;\mathrm{and}$$

$$\mathrm{\delta D}=a_{2}\times S+b_{2},$$

we obtain dependence of δD on δ^18^O, which corresponds to the linear equation in coordinates δ^18^O – δD:

$$\mathrm{\delta D}=a_{1}/a_{2}\left(\delta^{18}O–b_{2}\right)+b_{1}=7.9\left(\delta^{18}O\right)-2.3.$$

In the plot of δ^18^O – δD, the Black Sea waters compositions are located below the Global Meteoric Water Line (GMWL) [37], which characterizes the composition of precipitation (see Figure 6). The resulting relationship between δD and δ^18^O is parallel to GMWL and practically runs through the SMOW composition (δD = 0‰ and δ^18^O = 0‰) to the composition area of the Marmara and the Mediterranean Sea waters. Thus, the plot δD - δ^18^O shows that the entire range of oxygen and hydrogen isotope composition of the Black Sea water can result from mixing the Mediterranean Sea water with a hypothetical freshwater component (δ^18^O = −6.32‰ and δD = −52.1‰).

According to the water balance [5], freshwater input into the Black Sea is formed from the river runoff (V_r_ = 352 km^3^) and precipitation (V_p_ = 300 km^3^). Part of the water is lost through evaporation (V_e_ = 353 km^3^). Total volume of freshwater input to the sea (V_f_ = 299 km^3^) equals to

$$V_{f}=V_{r}+V_{p}-V_{e},$$

and isotope mass balance for the oxygen and hydrogen isotope composition of water can be described by equation

$$\delta_{f}V_{f}=\delta_{r}V_{r}+\delta_{p}V_{p}-\delta_{e}V_{e}.$$

Subscript r, p, e and f denote rivers input, precipitation, evaporation and fresh water input respectively. The solution of this equation with respect to δ_e_ allows us to calculate the average annual isotope composition of the evaporating component for which there exist no published data. Calculation of the oxygen and hydrogen isotope composition in evaporation is a difficult task, since δD and δ^18^O values are affected by the average annual temperature, humidity and wind stress, which determine the extent of their deviation from the equilibrium evaporation [11]. However, if the data on the mass balance of the freshwater input are consistent, then they can be used to calculate the average annual isotope composition of evaporation.

For the river runoff, we suggest that it is close to the averaged isotope data for 500 km of lower Danube River (δ^18^O = –9.73 ± 0.06‰, δD = −69.44 ± 0.81‰) [30]. Besides those for the Danube, there are no data available on the oxygen and hydrogen isotope composition in the mouths of rivers from the Black Sea catchment. δ^18^O and δD values of precipitation can be obtained using the database GNIP (Global Network of Isotopes in Precipitation, IAEA) when extrapolated to the point with coordinates 38° E, 43° N (approximate location of the stations in the center of the sea). Data from the GNIP database [35] show average annual δ^18^O and δD values for precipitation −7.7 ± 0.7‰ and −50 ± 6‰ (95% CL), respectively.

The calculated results are shown in Figure 6. The evaporation line connects integrated annual evaluation of evaporated moisture (δ^18^O = −10.9‰; δD = −67.6‰) with a point of freshwater input. The point of intersection with the line connecting the river water composition and the meteoric water is the freshwater composition resulted from mixing of river runoff and meteoric waters in the Black Sea. Its composition is changed under the influence of evaporation along the evaporation line to freshwater component of the Black Sea, which is mixed with LBC water, forming all water compositions of the Black Sea.

### Sulfur isotope composition of hydrogen sulfide in BCL

The distribution of sulfur isotope composition of hydrogen sulfide has not been considered previously taking into account the existence of an anaerobic zone with two water masses: the upper part stratified by density (depth < 1750 meters), and the lower part with homogeneous hydrophysical and hydrochemical parameters (the Bottom Convective Layer).

Hydrogen sulfide with the maximum ^34^S enrichment, δ^34^S (H_2_S) = −32.6‰, was discovered in the upper part of the anaerobic zone (depth 100–300 m) [13,14,28,38]. The value of δ^34^S (H_2_S) then decreased from −32.6 to −40.8‰ from the depth of the onset of H_2_S to below 300 m. Enrichment of δ^34^S (H_2_S) in the upper part of the anaerobic zone could be caused by an increase in sulfate reduction rates [15,39], and the quality and quantity of organic matter [40,41]. From 300 to 1500 m the values of δ^34^S (H_2_S) vary little, and on average are close to −40‰ [12,14,28]. Deeper than 1500 m, our data indicate that the values of δ^34^S (H_2_S) were not greater than −40.0‰ throughout the BCL. The average δ^34^S (H_2_S) for the two stations was not different. Near the bottom, at depths greater than 2000 m, the δ^34^S (H_2_S) value decreased from −40.0 to −41.0‰ (see Figure 2).

Decrease in δ^34^S (H_2_S) by about 1‰ in the near bottom area may result from the influence of hydrogen sulfide from pore water of sediments. Flux of hydrogen sulfide from sediments may be marked by an increase in its content in water-sediment interface. However, we did not observe the directional increase of hydrogen sulfide content in the near bottom area (see Tables 3, 4 and Figure 2). Variations of δ^34^S (H_2_S) values at the bottom water - pore water interface can only be found for stations 6, 7 and 8 [14,21,42] (see Figures 1 and 2). At the stations 6 and 7 (not shown), hydrogen sulfide flux was directed into the bottom water causing the increase of δ^34^S (H_2_S) by 0.7-0.8‰ at station 6 and by 2‰ at station 7. The data for δ^34^S (H_2_S) from pore water of sediments (depth 10–18 cm) in the western central part of the deep basin of the Black Sea (station 8, depth 2045 m) were close to δ^34^S (H_2_S) in the waters of the BCL (δ^34^S (H_2_S) = −40.4‰) (see Figure 2). In this case, an impurity of sulfide with identical δ^34^S (H_2_S) value cannot be defined in the near bottom water. Konovalov et al. [20] provided data on hydrogen sulfide content in the sediments and bottom water column at station 8–07, located about 20 miles southwest of stations 3426/100-50 (see Figure 1). Surface sediments of station 8–07 were presented by microlaminated oozes Unit 1. They had high hydrogen sulfide content, up to 1600 μM, which is four times higher than that in the overlying water. Flux of hydrogen sulfide from the sediment was result of some increase in the H_2_S content in the overlying bottom water.

According to our data, hydrogen sulfide from pore water of the deep basin sediments probably has no influence on the content of hydrogen sulfide in the water column. Moreover, in the near bottom waters of BCL, hydrogen sulfide has a light isotope enrichment of sulfur in comparison with dissolved hydrogen sulfide at depths from 1700–2000 m (see Figure 2). For the two stations under investigation, the average δ^34^S (H_2_S) value corresponded to −40.6 ± 0.4‰ for n = 29. Variation of sulfur isotope composition in hydrogen sulfide for the Black Sea anaerobic waters, based on the results of studies by different authors, is shown in Figure 2. Though the data are quite scattered they do not support an increase of δ^34^S (H_2_S) in the near bottom as observed by Neretin et al. [14].

### Variability of sulfur isotope composition of sulfate

Seawater sulfate is the source of hydrogen sulfide sulfur during the process of sulfate reduction in the anaerobic zone of the Black Sea. Previous data for the isotopic composition of sulfate sulfur showed that the deep part of the Black Sea is enriched δ^34^S (SO_4_) by about 2‰ [12]. However, those data are systematically lower by 2–3‰ relative to new data obtained by us (see Figure 2). The values of δ^34^S for sulfate in the Black Sea from Vinogradov et al. [16], Fry et al. [13] and Volkov et al. [43] were also somewhat lower than those obtained in the present study.

Sweeney and Kaplan [12] and Vinogradov et al. [16] reported the δ^34^S (SO_4_) value for California coastal water (19.70‰) and for Indian Ocean water (19.7‰), respectively. These data are provided as reference values for evaluation of analyses accuracy in different laboratories. Accepted data for sulfur isotope composition of sulfate has been 20‰ prior to the report of Rees et al. [44], which presented new data (20.99‰) on sulfur isotope composition of ocean water sulfate. Consequently, the only known δ^34^S (SO_4_) value for LBC water is not equal to 19.8‰ CDT [16], but it is at least close to 20.8‰ relatively CDT. Data in the VCDT scale may differ by 0.4‰ from that in the CDT scale [45]. Therefore sulfur isotope composition of sulfate in LBC inflow may be close to a modern sulfur isotope composition in ocean water [46].

Mediterranean water is the source of the LBC inflow. Böttcher et al. [47] provided data for isotope composition of sulfate sulfur 20.7‰ (VCDT) in surface water at station 973, located in the eastern Mediterranean. They also presented data for the reference material NBS 127, which was equal to 20.59 ± 0.08‰ (VCDT). These data are by 0.5‰ lower than the reference value [48]. In their short communication Neretin et al. [17] showed that down to the depth of 100 m in aerobic zone of the Black Sea the sulfur isotope composition in sulfate was nearly constant (from 20.5 to 20.7‰ VCDT). One can assume that these data were also obtained by M.Böttcher relative to NBS 127, which isotope composition of sulfur was equal to 20.59‰. Consequently, the sulfur isotope composition in sulfate of the Mediterranean water does not differ from the ocean water [46], the water of LBC and surface water from the eastern part of the Black Sea and is close to +21.1‰ in scale VCDT. Sulfate of the aerobic zone with a sulfur isotope composition of about +21.0‰ is not subjected to microbial reduction.

Based on 29 samples data (2 stations), the average isotope composition of sulfate sulfur deeper than 1250 m was +23.0 ± 0.2‰ (1σ). This was consistent with the data from the Bottom Convective Layer, and also at its boundary with the sediments. Enrichment of δ^34^S (SO_4_) is probably associated with sulfate reduction in the BCL itself.

### Sulfur isotope fractionation in the Black Sea

The observed fractionation of sulfur isotopes (ϵ) between sulfate and sulfide in the Black Sea water is close to 66.4‰. This is the highest value found for the modern anaerobic marine basins. By comparison, sulfur isotope fractionation in the Cariaco Basin is 54‰ [49], and in the Framvaren Fjord water it varies from 37 to 47‰ [50]. Large fractionation between sulfate and sulfide (greater than 47‰) can be explained by the processes of bacterial sulfate reduction at extremely slow microbial metabolism which can be attributed to limited availability and/or poor reactivity of organic substrate [41]. Sim et al. [41] found isotope fractionation of ϵ = 65.6% during sulfate reduction by bacterial culture *Disulfovibrio sp.* slow-growing on glucose. According to their study, isotopic fractionation in equilibrium between dissolved sulfate and sulfide can reach 68 ± 2‰ at 20°C.

There are no systematical studies of sulfate reduction rates with dependence on the season in the Black Sea. Fluxes of particulate organic carbon vary strongly with season and highest fluxes are found in summer and autumn [18]. The sulfate reduction rates in the water column and surface sediment may vary accordingly. The sulfur isotope composition of hydrogen sulfide and sulfate in the BCL was investigated at stations 3426 and 100–50 for different seasons. Significant differences in the distribution of sulfur isotopes in sulfate and hydrogen sulfide were not identified. We can assume that either the system (BCL) is not sensitive to seasonal change of organic matter fluxes, or our measurements too rough to distinguish seasonal changes in the system. BCL insensitivity to seasonal changes and the homogeneity of the distribution of hydrophysical and hydrochemical parameters can be explained by the low mass exchange with the sediments and overlying water column compared to the convective mixing time (40 years).

To estimate possible annual fluxes of mass exchange, it is necessary to consider the residence time of BCL. From the data [51], we estimated the volume of BCL below 1750 m as 57574 km^3^, and the area of the upper boundary as 187352 km^2^. Based on the model of Ivanov and Samodurov [52], the vertical velocity of water at the upper boundary of BCL was estimated as 6.8 × 10^−9^ m s^−1^. The upwelling of water at the upper boundary of BCL is provided by inflow of shelf modified Mediterranean waters in the same amount. Taking into account the area of the upper boundary and the velocity of upwelling, 40.2 km^3^ of water will leave the layer annually, and hence the same amount will be supplied. The time required for water renewal will be about 1430 years. These data almost coincide with the data of radiocarbon dating - 1500 years for the reservoir of water at the depth of 1400–2000 m [53]. These results do not contradict the exchange time of water (387 years) below the CIL [1]. According to the data [54], residence time of water increases exponentially with depth and is equal to 625 years at depth of 500 m.

A sulfide budget in the water column of the Black Sea was considered in [21]. Production of hydrogen sulfide occurs mainly in the lower part of water column (500–2200 m), and only about 10% is produced in the sediments. The residence time of the hydrogen sulfide in the water column is quite small and is estimated to be about 90–150 years. The residence time of sulfate in the water of the Black Sea is over 1000 years [4]. It strongly depends on the assumed value of LBC inflow which is the main supplier of sulfate into the Black Sea. With the accepted volume of LBC inflow close to 312 km^3^, the annual delivery of sulfate will be 884 Tg (29.5 mM × 312 km^3^). Only 8.4 Tg of sulfate is supplied by river runoff [55]. Based on the residence time in the Black Sea, sulfate is more conservative component than hydrogen sulfide.

Total sulfate inventory in BCL is about 98238 Tg (17.8 mM × 57574 km^3^). Besides the amount consumed for sulfate reduction in the water column of BCL, sulfate is supplied to sediments in the amount of 1.24 ± 0.47 Tg annually. This calculation is based on data on the pore profiles of sulfate from [23]. The average SO_4_ flux was obtained from six stations and was equal to 0.19 ± 0.07 mmol m^−2^ day^−1^. The total area of sediments below the depth of 1750 m was estimated as 187408 km^2^. The result of our calculations shows that annual amount of sulfate consumed in sediments would be less than 0.002% of the total amount of sulfate in the BCL. Sulfate flux through the upper boundary can be represented on the basis of constant BCL volume. If we consider annual removal of sulfate into the overlying water column only through the process of seawater upwelling and exclude diffusion transfer, then at the vertical velocity of upwelling 6.8 × 10^−9^ m s^−1^[52], 0.07% of total sulfate would be removed from the BCL.

Sulfate reduction of dissolved sulfate causes the liberation of H_2_S from the BCL. If no or very small externally derived SO_4_^2−^ input into the BCL compared to total amount of SO_4_^2−^ in it, this process can be referred as “closed” system even if it is clear that evolved H_2_S has left the BCL. If all liberated H_2_S is ^34^S depleted relative to SO_4_^2−^ at BCL, the ^34^S/^32^S of dissolved sulfate will increase because ^34^S-depleted H_2_S escapes the BCL. The magnitude of this effect will vary with the amount of H_2_S escaped according the mass balance. The process of Rayleigh distillation involves the continuous exchange and removal of each small portion of H_2_S. This description is more suitable for BCL. For a small amount of total H_2_S escaped there are no or little differences between mass-balance and Rayleigh calculations.

**Figure 7:**
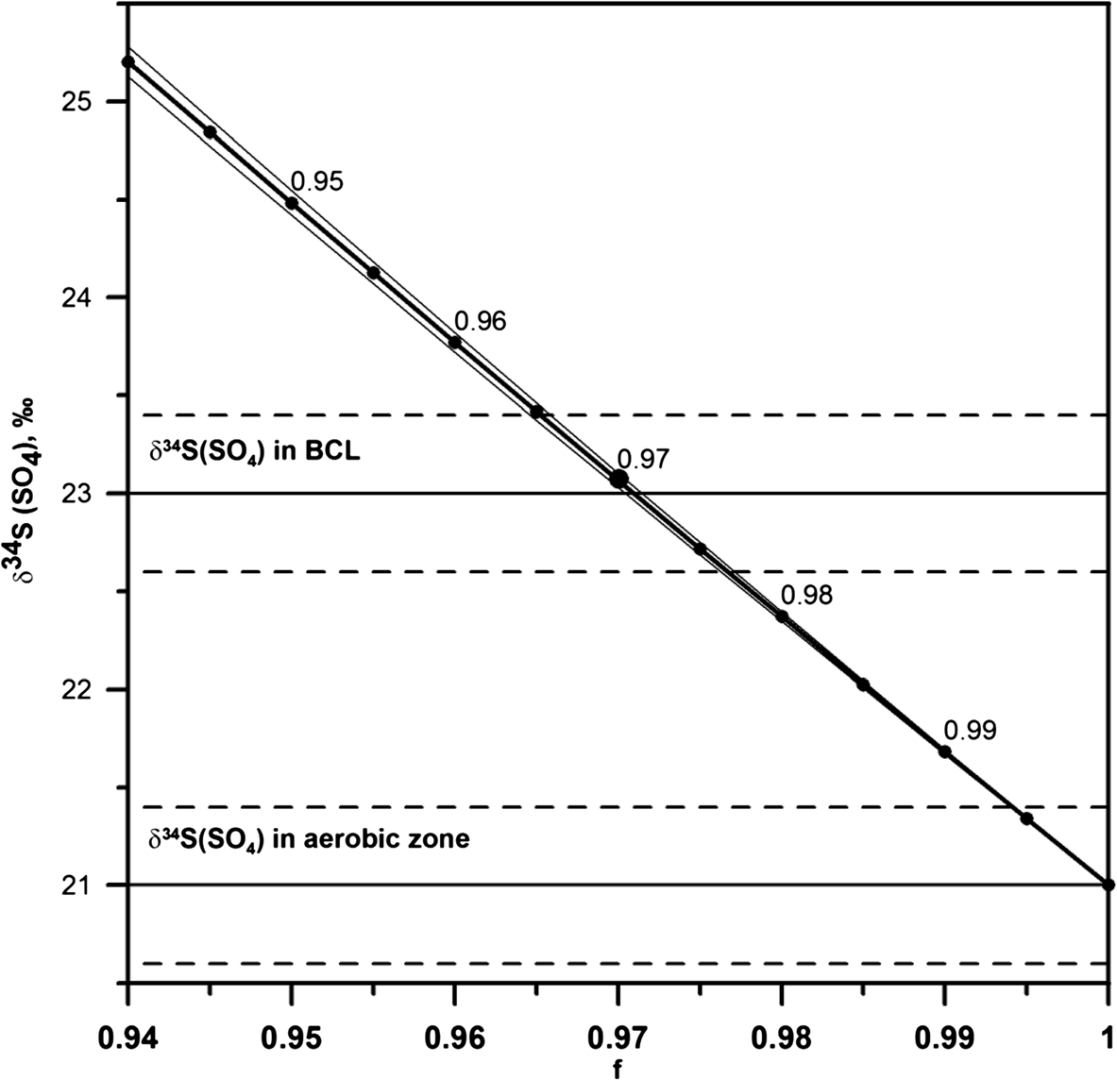
**Possible residual sulfate fraction in the BCL was calculated by Rayleigh distillation model assuming that the sulfur isotope composition in sulfate changes from +21 ± 0.4 (2σ) ‰ in aerobic zone to +23 ± 0.4 (2σ) ‰ in anaerobic one due to sulfate reduction.** Change of sulfate mass fraction loss is shown for fractionation factor α = 1.0664 ± 0.0012 (2σ) calculated as the average of all samples from BCL. It is also shown 2 σ confidence intervals for the average δ^34^S (SO_4_) values (dash line) and for the enrichment factor (thin lines).

To evaluate the loss fraction of sulfate due to sulfate reduction processes in the water column, we applied Rayleigh distillation model. This approach was repeatedly used earlier for estimating the fractionation factor [13,49,50]. The change in δ^34^S (SO_4_) is described by the Rayleigh distillation model, which relates the change in isotopic composition of sulfate sulfur (δ^34^S (SO_4_)_BCL_) relative to its initial composition (δ^34^S (SO_4_)_LBC_) with decrease of its fraction (f) (Figure 7):

$$\delta^{34}S\;{\left({\mathrm{SO}}_{4}\right)}_{\mathrm{BCL}}-\delta^{34}S\;{\left({\mathrm{SO}}_{4}\right)}_{\mathrm{LBC}}=\left(1-\alpha \right)\times \mathrm{Ln}\;f.$$

The initial value of δ^34^S (SO_4_)_LBC_ = +21‰ is taken as the isotope composition of sulfate sulfur in the LBC. A fractionation factor (α) between sulfide and sulfate in the BCL for the two stations is close to 1.0664 ± 0.0012 (2σ). This was obtained by averaging all data within the BCL. The calculation shows that increase in δ^34^S (SO_4_) from +21 ± 0.4 (2σ) to +23 ± 0.4 (2σ)‰ should be accompanied by a decrease of sulfate content by 3 ± 1 (2σ)%.

The decrease in the sulfate content relative to chloride in the deep waters of the Black Sea has been reported earlier by Kremling [22], Skopintsev [4], Bezborodov and Eremeev, [19], Jørgensen et al. [23] and others. According to the long-term observations, the average sulfate content in the aerobic zone of the Black Sea varies from 14.9 to 16.7 mM, and the SO_4_^2−^/Cl^−^ ratio is 0.1409 g g^−1^[4,19]. Sulfate-chloride ratio of ocean water varies in a range of 0.1393-0.1420, and this is close to the ratio in many inland seas [56]. This suggests that there are no anomalies of sulfate in the aerobic zone of the Black Sea relative to sulfate in open ocean seawater. With increasing depth in the anaerobic zone, sulfate content increases up to 17.8 mM (because salinity increases), but the SO_4_^2−^/Cl^−^ ratio goes down to 0.136-0.137 g g^−1^[4,19]). Loss of sulfate can be calculated by dividing SO_4_^2−^/Cl^−^ ratio in BCL by that in aerobic zone (0.137/0.141 = 0.97). This value corresponds to the loss of 3% of sulfate due to bacterial sulfate reduction. The coincidence of the results for the loss of sulfate shows that sulfate is consumed in the course of sulfate reduction processes in the BCL water column.

## Conclusions

New data on δ^18^O and δD values in water and sulfur isotope composition of hydrogen sulfide and sulfate in the Bottom Convective Layer have been presented for two stations located in the eastern central part of the Black Sea. Both stations have the same location but were sampled in different seasons: in August 2008 (station 3426) and in March 2009 (station 100–50).

The distribution of hydrogen and oxygen isotope composition of the Black Sea water was determined by mixing processes of two end-members: fresh water input and Mediterranean water from the Lower Bosporus Current. On the base of linear relationship of δ^18^O and δD versus salinity it was possible to obtain isotopic composition of fresh water input (δ^18^O = −6.32‰ and δD = −52.1‰) which includes the runoff and precipitation modified by evaporation. Using the known mass water balance for the Black Sea [5], isotopic composition of water for the Danube River (57% of total runoff) and IAEA data for annual precipitation, isotopic composition of evaporation (δ^18^O = −10.9‰; δD = −67.6‰) has been calculated. It was showed that annual δ^18^O and δD values were close to that of precipitation in cold months of the year from November to March. Comparison of δD distribution, which is a more sensitive parameter of water composition, revealed the differences exceeding 1‰ at water depth of the main picnocline (200–400 m) and in BCL, at 5 m above the bottom. Observed positive anomaly in δD distribution in the summer of 2008 versus spring 2009 might result from intrusion with significant fraction of shelf modified Mediterranean water [3] penetrated simultaneously into the picnocline and BCL.

For the two investigated stations the δ^34^S (H_2_S) distribution in BCL is homogenous with average value equal to 40.6 ± 0.5‰ (1σ). The average δ^34^S (H_2_S) in the BCL does not differ between summer −40.8 ± 0.5‰ and spring −40.5 ± 0.4‰. These sulfur isotope data are within the range of variations for the Black Sea water column −39.6 ± 1.3‰, determined previously by Neretin et al. [14]. In the near bottom area deeper than 2000 m, the average δ^34^S (H_2_S) gets more depleted and the average δ^34^S (H_2_S) in the BCL decreases to −41.0‰.

New data on sulfur isotope composition of sulfate from aerobic and anaerobic zones of the Black Sea have been obtained. The values of δ^34^S (SO_4_) are 2-3‰ higher than data published previously [12]. Sulfate of the aerobic zone with a sulfur isotope composition of about +21.0‰ and a SO_4_^2−^/Cl^−^ ratio that corresponds to sulfate of ocean water is not subjected to microbial reduction. Data on 29 samples from 2 stations showed that the average sulfur isotope composition of sulfate below 1250 m was +23.0 ± 0.2‰ (1σ). This value did not depend on the season of observation and remained constant within the reproducibility of analysis. The fractionation factor (α) between sulfide and sulfate in the BCL for the two stations was close to 1.0664. Application of Rayleigh distillation model shows that the δ^34^S (SO_4_) increase from +21 to +23‰ due to sulfate reduction was accompanied by a decrease in the amount of marine sulfate by 3%. Fractionation of sulfur isotopes in the Black Sea is the highest (66%) found for the contemporary anaerobic marine basins. Such fractionation might be a result of a very low rate of sulfate reduction limited by quality and quantity of organic matter.

## Abbreviations

LBC: Lower bosporus Current; BCL: Bottom convective layer; CIL: Cold intermediate layer; GMWL: Global Meteoric Water Line; RSD: Relative standard deviation; SRR: Sulfate reduction rate.

## Competing interests

The authors declare that they have no competing interests.

## Authors’ contributions

AVD conceived of the study, participated in its design and coordination, interpreted the results and drafted the manuscript. EOD carried out the stable isotope analyses, interpreted the results and helped to draft the manuscript. TPD carried out sample collection and chemical preparation for isotope measurement. NMK carried out sample collection and onboard sample preparation for isotope measurement. MNR carried out sample collection and onboard sample preparation for isotope measurement, helped to draft the manuscript. SAK carried out sample preparation for isotope measurement. EVY carried out sample collection and helped to draft the manuscript. All authors read and approved the final manuscript.
